# Protein-protein interaction analysis in crude bacterial lysates using combinational method of ^19^F site-specific incorporation and ^19^F NMR

**DOI:** 10.1007/s13238-016-0336-8

**Published:** 2016-10-27

**Authors:** Dong Li, Yanan Zhang, Yao He, Chengwei Zhang, Jiefei Wang, Ying Xiong, Longhua Zhang, Yangzhong Liu, Pan Shi, Changlin Tian

**Affiliations:** 1 Hefei National Laboratory for Physical Science at the Microscale & School of Life Science, University of Science and Technology of China, Hefei, 230026 Anhui China; 2 School of Chemistry, University of Science and Technology of China, Hefei, 230026 Anhui China

**Dear Editor,**

Protein-protein interactions (PPI) are essential for a variety of cellular functions. Many PPI analyses were conducted *in vitro*, using purified proteins. In this report, the unnatural amino acid tfmF was site-specifically incorporated into several different sites of two Phox-Bem1 (PB1) domains from two mitogen activated protein kinases (MEKK3 and/ or MEK5) in the *E. coli* cells. Solution NMR ^19^F chemical shift and side chain relaxation analysis demonstrated that MEKK3-PB1-I57, MEKK3-PB1-F77, and MEK5-PB1-I70 sites were located in the interaction interface of the MEKK3/MEK5 complex, which was consistent with the crystal structure of MEKK3-PB1/MEK5-PB1 complex. Furthermore, crude lysates from *E. coli* cells with co-expressed tfmF incorporated MEKK3-PB1 and MEK5-PB1 were applied for ^19^F NMR analysis. The successful implementation of *in situ* PPI analysis using the combinational method of site-specific tfmF incorporation and ^19^F NMR demonstrated that this method could be a valuable general method for conformation and function studies of soluble multi-domain proteins or protein complexes in bacterial crude lysate, without procedures of protein purification.

Protein-protein interactions (PPI) play essential roles in cellular functions, such as DNA transcription, signal transduction, or cytoskeleton formation. Currently, a variety of techniques, including co-immunoprecipitation, isothermal titration calorimetry, and surface plasma resonance are frequently applied for PPI studies (Syafrizayanti et al., 2014). However, these methods can only provide the overall interaction pattern or internal motion of purified protein complexes, and have many limitations such as low specificity, high background or false positives (Syafrizayanti et al., 2014). Structure determination methods (such as X-ray crystallography and electron cryo-microscopy) can precisely illustrate protein interaction interface, while these structural methods require high concentration of purified proteins.

Recently, it has been reported that the cytoplasmic environment might have profound effects in regulating protein–protein and/or protein–ligand interactions that were hardly observed *in vitro* (Smith et al., 2014). The crucial difference between *in vivo* and *in vitro* conditions lies in the high environmental concentration of diverse macromolecules, which is approximately 200 mg/mL in the eukaryotic cytoplasm and more than 400 mg/mL in prokaryotes (Mika and Poolman, 2011). Traditional *in vitro* biochemical studies of proteins were conducted in dilute solution with low macromolecular concentration (~10 mg/mL), which might not reveal protein-protein interaction or its mechanism in high fidelity.

Solution nuclear magnetic resonance (NMR) is powerful for PPI analysis and recently it has been applied to analyze protein conformation changes in living cells (Hansel et al., 2014). However, the traditional *in-cell* NMR method was hindered by low signal sensitivity and complicated resonance assignment of proteins *in vivo* (Hansel et al., 2014). At the same time, the solution NMR signals of large size proteins were very weak in intensity, with broad line width, due to slow global correlation time and rapid nuclei spin relaxation rate. Consequently, NMR resonance assignments of uniformly isotope labeled proteins will be very laborious due to strong overlaps in peaks with broad line width and low intensity. Alternatively, site-specific ^19^F incorporation and ^19^F NMR could provide a tool to implement PPI analysis *in situ* or *in vivo*. In the past decades, ^19^F NMR has been widely used for protein dynamic conformation changes and functional studies (Guo et al., 2015; Shi et al., 2011). Different from traditional multiple-site ^19^F incorporation through growing bacteria in media containing the ^19^F-aromatic residues (Lee et al., 2000), the ^19^F-containing unnatural amino acids could implement site-specific ^19^F labeling, resulting in the straight-forward resonance assignment (Hammill et al., 2007; Li et al., 2010). The trifluoromethyl phenylalanine (tfmF) was successfully used for analyses of membrane protein’s conformation changes, dynamics and functions (Shi et al., 2012). Additionally, the fast rotational motion of the CF_3_- group in tfmF leads to a sharp single peak, which makes the tfmF-^19^F NMR method ideal for protein complex studies in high crowding conditions, such as cell lysate or cellular environment. Therefore, combinational application of the tfmF incorporation and ^19^F NMR for *in situ* PPI analysis would be immensely valuable, not only for PPI mechanism studies, but also for PPI drug design with high specific and potent therapeutic principles against many diseases.

Here, the ^19^F-NMR PPI analysis in the native cellular environment was exemplified using the Phox and Bem1 (PB1) domains from two mitogen-activated protein kinases (MAPKs): MEKK3 and MEK5 (Drew et al., 2012). The MEKK3-PB1 domain (type II group) contains a positively charged basic cluster in the front end, whereas the MEK5-PB1 domain (type I group) contains a negatively charged acidic OPCA motif in the back end. The electrostatic interactions were known to be the major force for heterodimer formation between the type II MEKK3-PB1 and type I MEK5-PB1 in a front-to-back manner (Hu et al., 2007). In this report, the unnatural amino acid ^19^F-tfmF was incorporated into several sites of MEKK3-PB1 and MEK5-PB1, respectively. Then, ^19^F NMR chemical shift and relaxation data were obtained to analyze the interaction interfaces between MEKK3-PB1 and MEK5-PB1. The ^19^F chemical shift perturbations of residues in the interfacial region of MEKK3-PB1/MEK5-PB1 complex in crude bacterial cell lysates (without protein purification) were observed to be consistent not only with the ^19^F chemical shift data of the purified protein complex, but also with the crystal structure of the MEKK3-PB1/MEK5-PB1 complex, which strongly indicated the validity of the proposed general method of ^19^F-tfmF/^19^F-NMR for *in situ* PPI analysis.

As shown in Fig. 1A, four residue sites (MEKK3-I57, MEKK3-F77, MEK5-I70 and MEK5-F41) were selected for site-specific tfmF incorporations. Size-exclusion chromatography (SEC) was applied to verify the complex formation between MEKK3-PB1-I57tfmF and MEK5-PB1-I70tfmF. In the SEC diagram, the earlier retention time of the MEKK3-PB1-tfmF/MEK5-PB1-tfmF than the MEKK3-PB1 or MEK5-PB1 indicated the stable complex formation (Fig. 1B). Single band in SDS–PAGE of purified MEKK3-PB1-I57tfmF and/or MEK5-PB1-I70tfmF with Ni^2+^-NTA affinity chromatography demonstrated a good purity of these proteins (Fig. 1B, inset). MEKK3-PB1 and MEK5-PB1 were co-expressed using plasmid pETDuet-1 for site-specific tfmF-incorporation, and were co-purified using Ni^2+^-NTA affinity chromatography (Fig. 1B, lane 1). Minor migration difference between the two bands was observed for ^19^F-MEK5-PB1 (lane 2) or ^19^F-MEKK3-PB1 (lane 3) (Fig. 1B).

**Figure 1:**
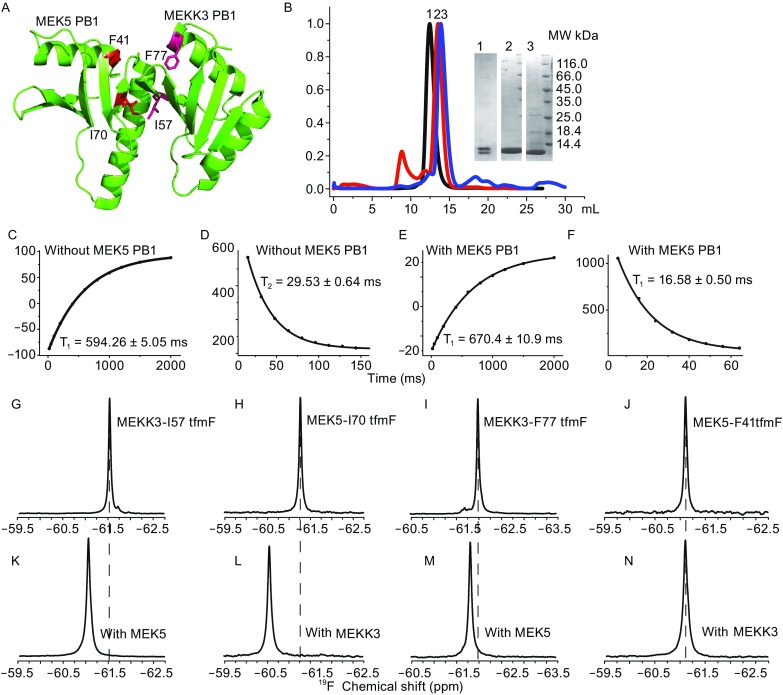
^**19**^ **F chemical shift perturbations and relaxation values of site-specific tfmF incorporation in purified MEKK3-PB1 or MEK5-PB1**. (A) Stereo ribbon drawing of the tertiary structure of the MEKK3-PB1 and MEK5-PB1 complex (PDB Number: 2O2V). The site-specific ^19^F incorporation sites (MEKK3-I57, MEKK3-F77, MEK5-I70, MEK5-F41) were coloured magenta and cyan in MEKK3-PB1 and MEK5-PB1 respectively. The figure was prepared using pymol. (B) Size exclusive chromatography of MEKK3-PB1 (blue), MEK5-PB1 (red), MEKK3-PB1/MEK5-PB1 complex (black) with tfmF incorporations. SDS–PAGE of MEKK3-PB1 (lane 3), MEK5-PB1 (lane 2), and co-purified MEKK3-PB1/MEK5-PB1, all with ^19^F incorporation (lane 1). Side chain longitudinal T_1_ (C and E) and transverse T_2_ (D and F) relaxation analysis of ^19^F site-specifically incorporated at the F77 site of MEKK3, in the absence or presence of MEK5-PB1 domain. One-dimension ^19^F spectra of tfmF incorporated MEKK3-PB1 domain in the absence (G and I) or presence (K and M) of the MEK5-PB1 domain. One-dimension ^19^F spectra of tfmF incorporated MEK5-PB1 domain in the absence (H and J) or presence (L and N) of the MEKK3-PB1 domain

To reveal motional properties of the tfmF-incorporation site and details of protein–protein interactions, both ^19^F longitudinal (T_1_) and transverse (T_2_) relaxation values of proteins with incorporated ^19^F-tfmF were measured. Here, the ^19^F T_1_ and T_2_ relaxation values of MEKK3-PB1-F77tfmF in the absence or presence of wild-type MEK5-PB1 were shown as Fig. 1C–F. Upon addition of MEK5-PB1, the T_1_ relaxation value of MEK50-PB1-F77tfmF was observed to increase (Fig. 1C and 1E), whereas the T_2_ relaxation value decreased (Fig. 1D and 1F). The ^19^F relaxation values of the four tfmF incorporation sites (MEKK3-PB1-F77tfmf/MEK5-PB1, MEKK3-PB1-I57tfmF/ MEK5-PB1, MEKK3-PB1/ MEK5-PB1-I70tfmF, MEKK3-PB1/MEK5-PB1-F41tfmF) were shown in both Fig. 1 and Table S1 (supporting information). The pronounced decrease in T_2_ values in the presence of another domain could be attributed to the decreased global motion with increased molecular size or restrained internal motions (Palmer, 1993). Considering the halved global correlation time for the formation of MEKK3-PB1/MEK5-PB1 complex (the almost doubled molecular weight), the decreased relaxation data demonstrated the formation of a stable complex between MEKK3-PB1 and MEK5-PB1.

To investigate the PPI interface between MEKK3-PB1 and MEK5-PB1, *in vitro* ^19^F chemical shift of MEKK3-PB1-I57tfmF and MEKK3-PB1-F77tfmF were acquired in the absence or presence of the wild-type MEK5-PB1. Similarly, the ^19^F chemical shift of MEK5-PB1-I70tfmF and MEK5-PB1-F41tfmF were collected in the absence or presence of the wild-type MEKK3-PB1. Pronounced ^19^F chemical shift changes in the absence and presence of wild-type MEK5-PB1 (or MEKK3-PB1) were observed for MEKK3-PB1-I57tfmF, MEK5-PB1-I70tfmF and MEKK3-PB1-F77tfmF (Fig. 1G, 1K, 1H, 1L, 1I and 1M). The tertiary structure of the MEKK3-PB1 (PDB Number: 2C60), MEK5-PB1 and MEKK3-PB1/MEK5-PB1 complex (PDB Number: 2O2V) in PDB did not show pronounced structure variations in these sites after complex formation. The observations of chemical shift changes indicated that the sites I57 and F77 of MEKK3-PB1 and I70 of MEK5-PB1 were located in the interaction interface of the MEKK3-PB1/MEK5-PB1 complex. However, no obvious chemical shift changes were observed for MEK5-F41tfmF (Fig. 1J and 1N), which might be away from the PPI interface (the chemical shift values of ^19^F site-specific-labeled residues were presented in Table S1). The observations of chemical shift changes upon protein interaction were consistent with the three-dimensional crystal structure of the MEKK3-PB1 and MEK5-PB1 complex (PDB 2O2V).

To implement the *in situ* PPI analysis, the combinational method of tfmF-incorporation and ^19^F-NMR was also applied. In physiological cytosolic conditions, proteins are known to stay in a highly crowded environment with vast of non-specific interactions. Therefore, the conventional *in vitro* protein–protein interaction mode might not represent the real situation in the physiological environment. Because the cell lysate composed of mixture of soluble biomolecules in the host cells, the PPI analysis in cell lysates could contain the vast varieties of proteins in the crowded environment, avoiding perturbations of PPI during the protein purification procedures. To implement the PPI analysis between MEKK3-PB1 and MEK5-PB1 in crude lysates, the two proteins were co-expressed in *E. coli* and the crude cell lysates were prepared as shown in Fig. 2A. Due to the pronounced chemical shift changes in purified MEKK3-PB1-I57tfmF and purified MEK5-PB1-I70tfmF, these two sites were selected for double site-specific labeling. One-dimension ^19^F spectra of the purified sample or crude lysate sample containing MEKK3-PB1-I57tfmF are shown in Fig. 2B and E. Only one ^19^F peak was observed for both the purified MEKK3-PB1-I57tfmF and the crude lysate sample, whereas the line width of the ^19^F-signal from the crude lysate sample was broader, obviously due to the crowding cellular environment, presence of non-specific protein interactions or chemical transient interactions in the crude lysate (Smith et al., 2014; Latham and Kay, 2013). The ^19^F NMR spectra of MEK5-PB1-I70tfmF in the purification buffer and crude lysate are shown in Fig. 2C and F, with an increased line-width for MEK5-PB1-I70tfmF in the crude lysate. For the samples of two tfmF-incorporated proteins, two peaks were observed for the co-purified sample of MEKK3-PB1-I57tfmF and MEK5-PB1-I70tfmF. As shown in Fig. 1G, K, H, and L, the ^19^F chemical shifts of MEKK3-PB1-I57tfmF and MEK5-PB1-I70tfmF were shifted downfield upon protein interaction. According to the ^19^F chemical shift values (Table S1) with single site labeling, the right peak in Fig. 2D could be assigned to MEKK3-PB1-I57tfmF, whereas the left was assigned to MEK5-PB1-I70tfmF. A significant shift of the ^19^F signal of MEKK3-PB1-I57tfmF and MEK5-PB1-I70tfmF were observed upon the presence of partner proteins of the complex, or in the presence of specific protein–protein interactions.

**Figure 2:**
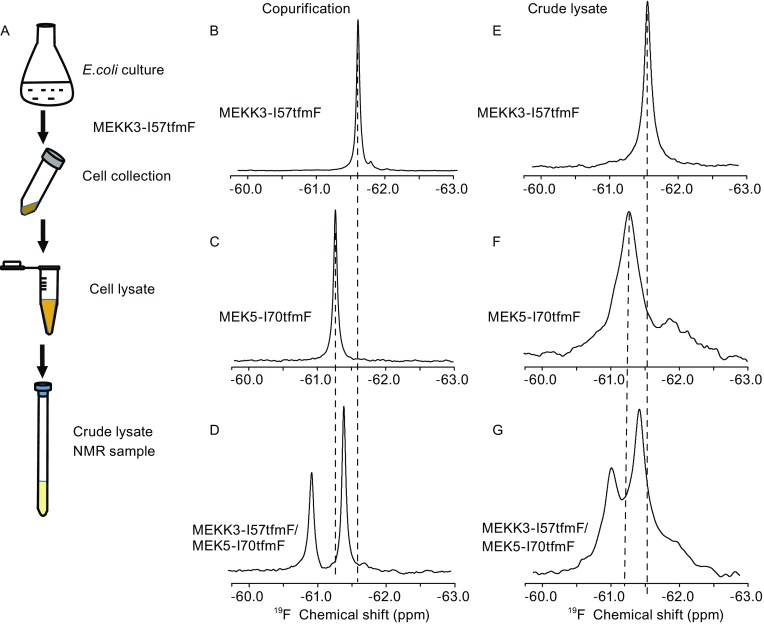
**One dimensional** ^**19**^ **F NMR spectra of MEKK3-PB1 or MEK5-PB1 in crude lysates with double site-specific tfmF incorporation**. Procedure of crude lysate sample preparation (A), One-dimension ^19^F spectra of purified MEKK3-PB1-I57tfmF (B), MEK5-PB1-I70tfmF (C), and co-expressed MEKK3-PB1-I57tfmF and MEK5-PB1-I70tfmF complex (D). One-dimension ^19^F spectra of MEKK3-PB1-I57tfmF (E), MEK5-PB1-I70tfmF (F), and co-expressed MEKK3-PB1-I57tfmF and MEK5-PB1-I70tfmF complex in bacteria crude lysate (G)

Additionally, different from ^19^F signals of the co-purified sample, two wider ^19^F signals were shown in Fig. 2G as a result of co-expressed proteins in crude lysate samples. The ^19^F NMR signals from crude lysates were much broader, due to molecular crowding or weak transient interactions in the crude lysate (Smith et al., 2014; Latham and Kay, 2013). Compared with solution NMR data of purified co-expression MEKK3-PB1 and MEK5-PB1, the crude lysate data illustrated that ^19^F chemical shift values of residues in crude lysate were influenced by the ubiquitous nature of weak, non-specific interactions in cells, which retarded the rotational motion of soluble proteins and the exchange dynamics. The increased line width of 1D ^19^F NMR spectra in the crude lysate sample presented the physiological environment of cell plasma.

Referring to the observed chemical shift perturbations of MEKK3-PB1-I57tfmF/ MEK5-PB1-I70tfmF in the *in vitro* PPI studies (Figs. 1G–N and 2B–D), the observed ^19^F chemical shift perturbations of the *in situ* PPI studies (Fig. 2E–G) verified the existence of protein interactions between MEKK3-PB1 and MEK5-PB1 in the *E. coli* cytosols, even in the presence of extensive, non-specific macromolecular interactions in cell lysate. For the MEKK3-PB1/MEK5-PB1 protein complex, the physicochemical mechanisms governing macromolecular assembly in the cell must be similar as those in cell extracts (Luh et al., 2013). At the same time, the increased line widths of ^19^F NMR signals of proteins in crude lysate implied the availability of many non-specific interactions with the target proteins, through some universal mechanisms like hydrogen bonds, charge-charge interactions, or random collisions in the cellular environment.

Normally, more than one condition could lead to the ^19^F chemical shift changes of the tfmF incorporation site: the localization in the PPI interface, or allosteric conformational changes after protein-protein interaction. Nevertheless, the ^19^F-tfmF chemicals shifts could represent the availability of protein-protein interactions, in cell lysate or other in-cell mimic conditions. Of course, multiple site incorporations of tfmF and ^19^F-NMR will be required to reflect the uniform changes of the sample conditions, *e.g.* acidification, viscosity changes or protein degradation. In this report the ^19^F-spectra of MEK5-F41tfmF (Fig. 1J and 1N) were working as the control to reflect the macro-scale condition changes.

To distinguish the PPI interface or the allosteric conformation changes, further ^19^F-detected relaxation analysis should be conducted. For the residue sites in the PPI interface, not only the ^19^F chemical shift changes were expected, but also variations of the T_1_ relaxation (spin-lattice), T_2_ relaxation (spin-spin diffusion) could be observed. However, for the allosteric conformation changes, the conformational exchange (τ_ex_) and T_2_ relaxation exchanges could be observed.

In summary, combinational method of site-specifically incorporation of the unnatural amino acid tfmF into proteins and ^19^F NMR could be a reliable method for PPI analysis in cellular cytosols, taking advantage of no natural ^19^F background signals from cellular molecules. At the same time, the tfmF incorporations at two residue sites using the pET-Duet plasmids in this report provided a general method for *in situ* PPI analysis between two tfmF-incorporated proteins. Therefore, conformational and functional studies of other soluble proteins (enzymes, receptors), or interaction interfaces analysis of two proteins in a complex in crude cell lysates could be implemented using the combinational method of site-specific ^19^F incorporation and ^19^F NMR.

## Electronic supplementary material

Below is the link to the electronic supplementary material.

Supplementary material 1 (PDF 198 kb)
