# Erratum to: Protein-protein interaction analysis in crude bacterial lysates using combinational method of ^19^F site-specific incorporation and ^19^F NMR

**DOI:** 10.1007/s13238-017-0389-3

**Published:** 2017-03-10

**Authors:** Dong Li, Yanan Zhang, Yao He, Chengwei Zhang, Jiefei Wang, Ying Xiong, Longhua Zhang, Yangzhong Liu, Pan Shi, Changlin Tian

**Affiliations:** 1 Hefei National Laboratory for Physical Science at the Microscale School of Life Science, University of Science and Technology of China, Hefei, 230026 China; 2 School of Chemistry, University of Science and Technology of China, Hefei, 230026 China

## Erratum to: Protein Cell 2017, 8(2):149–154 DOI 10.1007/s13238-016-0336-8

In the original publication of this article the correspondence authors’ e-mail has been missed out.

The missing e-mail addresses are provided in this erratum.

