# Supplementary material for: Protein-protein interaction analysis in crude bacterial lysates using combinational method of 19F site-specific incorporation and 19F NMR
Source: Protein Cell. 2016 Oct 27;8(2):149–54. doi: 10.1007/s13238-016-0336-8 (PMC5291773; doi:10.1007/s13238-016-0336-8)
Supplement: Supplementary file 1 — Supplementary material 1 (PDF 198 kb) [file 13238_2016_336_MOESM1_ESM.pdf]

Protein-protein interaction analysis in crude bacterial lysates using combinational method of ^{19}F site-specific incorporation and ^{19}F NMR

Dong Li[#], Yanan Zhang[#], Yao He, Chengwei Zhang, Jiefei Wang, Ying Xiong, Longhua Zhang, Pan Shi*, Changlin Tian*

Hefei National Laboratory for Physical Science at the Microscale & School of Life Science, University of Science and Technology of China, Hefei, Anhui 230026, P. R. China

Supplemental Materials

Plasmid construction and site-specific mutagenesis

The amplified MEKK3-PB1 and MEK5-PB1 coding sequence were inserted into the pBAD28 vector (Invitrogen, Thermo Fisher Scientific, USA) between the *Nde I* and *Xho I* restriction endonuclease sites. Two residue sites (I57, F77) of MEKK3 and two residue sites (F41, I70) of MEK5 were selected, and codons corresponding to these sites were mutated to the amber stop codon TAG using a standard PCR-based mutagenesis method. Mutagenesis was confirmed by DNA sequencing. The mutated pBAD28 plasmid and another essential plasmid, pDule-tfmf (containing the coding sequence for $tRNA_{CUA}$ and tfmF-specific aminoacyl-tRNA synthetase)(Hammill et al., 2007) were co-transformed in *E. coli* host Top10 cells for further protein expression.

For the co-expression of MEKK3-PB1 TAG and MEK5-PB1 TAG mutants, another plasmid named pETDuet-1 (Invitrogen, Thermo Fisher Scientific, USA) was used, which contained two multiple cloning sites and a ribosomal binding site. The MEKK3-PB1 DNA fragment was inserted into the pETDuet-1 plasmid using *BamH I* and *Not I* restriction enzymes using the standard PCR method. The DNA fragment of MEK5-PB1 was then cloned between the *Nde I* and *Xho I* restriction sites. The corresponding TAG mutants of two DNA fragments were performed using the two-step PCR scheme. The constructed plasmid was co-transformed with pDule-tfmF into *E. coli* host Top10 cells.

Site-specific tfmF incorporation and protein purification

The transformed bacteria were incubated in LB medium containing 1 mM tfmF at

37°C (with shaking at 225 rpm) with 100 μg/mL ampicillin and 15 μg/mL tetracycline. The transformed bacteria were incubated in LB medium overnight, and then transferred into fresh 2x YT medium containing 1 mM tfmF at 37°C in presence of 100 μg/mL ampicillin and 15μg/mL tetracycline. Expression of the target protein was induced using 0.2% arabinose when the OD_{600} reached 1.0. After 4 h, bacteria were harvested by centrifugation at 3700g for 20 min at 4°C (Beckman Coulter X-15R). The pellet was collected and suspended in 40 mL lysis buffer (20 mM Tris, 500 mM NaCl, pH 8.0), and then probe-sonicated (VC500, Sonics and Materials, Danbury, CT) on ice for a total of 10 min (power level, 30%; 3.0 s pulse on and 5.0 s pulse off). The cell lysate was centrifuged at 23,000 g for 20 min at 4°C (Hitachi Himac Centrifuge, CR21GII), after which the supernatant was collected and mixed with preprocessed 3 mL Ni^{2+}-NTA resin (QIAgen, Valencia, CA). The mixture was mixed at 4°C for 40 min, followed by packing onto a gravity-flow column (BIO-ARD, Hercules, CA). The impurities were washed out by a series of imidazole gradients, and the target protein was then eluted using elution buffer (50 mM Tris, 200 mM NaCl, 250 mM imidazole, pH 8.0).

The concentration of purified target protein was determined using OD_{280} measurements, and analyzed by standard SDS–PAGE (15%, w/v). The purified target protein was concentrated to a final volume of 450 μL using an Amicon Ultra-15 device (3000 MWCO, Millipore). Then, 50 μL D_2O was added to the sample to a 10% (v/v) final concentration before solution NMR analysis.

Crude lysate sample preparation

Cell cultures (about 200 mL for co-expressed MEKK3 PB1 and MEK5 PB1) were centrifuged at 2000g for 20 min at 4°C (Beckman Coulter X-15R). The pellets were resuspended in 50 mL LB media and centrifuged at 2000g for 20 min at 4°C (Beckman Coulter X-15R) for several cycles to remove the free tfmF compound in the media. The cells were resuspended in 2.5 mL of lysis buffer (20 mM Tris, 300 mM NaCl, pH 8.0) and sonicated on ice by sonication (VC500, Sonics and Materials, Danbury, CT) for a total of 10 min (power level, 30%; 3.0 s pulse on and 5.0 s pulse off). Supernatants were collected after centrifugation at 123,000 g for 20 min at 4°C (Hitachi Himac Centrifuge, CR21GII). Then the supernatant was flowed-through from the PD-10 desalting columns (GE Healthcare Life Sciences). Then, the supernatant was concentrated to a final volume of 450 μL. D_2O was added to the sample to a 10% (v/v) final concentration before ^{19}F solution NMR analysis.

^{19}F chemical shift and side chain relaxation analysis

All one-dimension ^{19}F NMR spectral measurements were performed at 293 K on an Agilent 500 MHz spectrometer equipped with a HFT probe, and the observation channel was tuned to ^{19}F (470.2 MHz) with 1024 free induction decay (FID) accumulations in every 4 s recycling delay. One-dimensional ^{19}F spectra were acquired with a one pulse program with a 90° pulse width of 12.7 μs and power at 57 w. The spectra width (SW) was 60 ppm and the offset was set at -62 ppm. ^{19}F chemical shifts were referenced to an external standard tfmF (-62.38 ppm), and the chemical shift of tetramethylsilane(TMS) was calibrated to 0 ppm. The data were processed with an exponential window function (LB =15 Hz) and plotted using

ACD/NMR Processor Academic Edition software (ACD/Labs). The spectra were collected at 293K.

A pulsed field gradient-based longitudinal eddy delay pulse sequence was applied to measure the relaxation time of proteins. The experiments were conducted at 298 K, in a 500 MHz Agilent spectrometer. Side chain ^{19}F T_1 relaxation data were collected with eight delay times (50, 100, 200, 400, 600, 800, 1000, 1200, 1500, and 2000 ms) using a standard 1D inverse recovery pulse sequence. The magnetization Mz was inverted by a 180° pulse. Waiting a delay τ, the amount of z-magnetization was measured using a 90° pulse. 64 scans were accumulated for one-dimensional ^{19}F chemical shift analysis and 256 scans were accumulated for T_1 data acquisition.

According to Bloch function, the dependence of the z-magnetization on τ is given by:

$$Mz(\tau) = M_0(1 - 2e^{-\tau/T1}) \quad (1)$$

M_0 is the equilibrium value of the z-magnetization.

Resonance intensities in relaxation experiments were measured and fit to an exponential function.

$$y = y_0 + Ae^{-x/t} \quad (2)$$

The Carr-Purcell-Meiboom-Gill sequence (CPMG) experiment allows measure transverse or spin-spin T_2 relaxation times of any nucleus. Side-chain ^{19}F T_2 transverse relaxation data were collected with ten delay durations (8, 16, 32, 48, 64, 80, 96, 112, 144, and 160 ms) using a standard 1D Carr-Purcel-Meiboom-Gill (CPMG) pulse sequence. The intensities of the series of peaks were regressed for transverse relaxation values for tfmF labeled MEKK3-PB1 or MEK5-PB1. Resonance intensities

in CPMG experiments were fit to an exponential function. The peak intensity values were analyzed and plotted using OriginPro 8.0.

$$y = y_0 + Ae^{-x/t} \quad (2)$$

In the measurement of the T_2 relaxation times, the magnetic field inhomogeneities (T_2^{inh}) must be considered:

$$1/T_2^* = (1/T_2) + (1/T_2^{inh}) \quad (3)$$

The experimental half-height line-width (d) of a given resonance is directly related to T_2^* by the equation.

$$d = 1/(\pi * T_2^*) \quad (4)$$

To estimate the T_2^* value of crude lysate sample the line width was read using ACD/Lab software.

Supplemental References

Hammill, J.T., Miyake-Stoner, S., Hazen, J.L., Jackson, J.C., and Mehl, R.A. (2007). Preparation of site-specifically labeled fluorinated proteins for 19F-NMR structural characterization. Nat Protoc 2, 2601-2607.

Table S1. ^{19}F side-chain chemical shifts, side chain T_1 and T_2 relaxation times of MEKK3 PB1 with or without MEK5 PB1; ^{19}F side-chain chemical shifts, side chain T_1 and T_2 relaxation times of MEK5 PB1 with or without MEKKK3 PB1.

Residue	Parameter	*in vitro* side chain ^{19}F		Crude lysate side chain ^{19}F	
		W/O MEK5 PB1 WT	With MEK5 PB1 WT	W/O MEK5 PB1 WT	With MEK5 PB1 WT
MEKK3-I57	Chemical shift (ppm)	-61.59	-61.11	-61.55	-61.14
	T_1 (ms)	964.22 ±5.34	672.65 ±6.59		
	T_2 (ms)	34.41 ±0.44	9.42 ±0.12		
	Calculated T_2 (ms)	10.72	6.74	6.91	4.32
	Line width (Hz)	29.69	47.2	46.06	73.73
MEKK3-F77	Chemical shift (ppm)	-62.02	-61.85		
	T_1(ms)	594.27 ±5.05	670.41±10.98		
	T_2(ms)	29.54 ±0.64	16.58±0.51		
	Calculated T_2 (ms)	10.40	7.72		
	Line width(Hz)	30.59	41.23		
		W/O MEKK3 PB1 WT	With MEKK3 PB1 WT	W/O MEKK3 PB1 WT	With MEKK3 PB1 WT
MEK5-I70	Chemical shift (ppm)	-61.31	-60.59	-61.27	-60.71
	T_1(ms)	1137.09±49.51	768.12±21.34		
	T_2(ms)	18.2 ±0.28	8.93±0.27		
	Calculated T_2 (ms)	8.89	6.39	2.69	3.6
	Line width(Hz)	35.82	49.82	118.23	88.39
MEK5-F41	Chemical shift (ppm)	-61.16	-61.16		
	T_1(ms)	792.92±17.98	1009.6±27.6		
	T_2(ms)	23.27±0.83	9.97 ±0.45		
	Calculated T_2 (ms)	8.93	6.22		
	Line width(Hz)	35.65	51.14		
